# Blood-based immunoassay of tau proteins for early diagnosis of Alzheimer's disease using surface plasmon resonance fiber sensors

**DOI:** 10.1039/c7ra11637c

**Published:** 2018-02-19

**Authors:** Truong Thi Vu Nu, Nhu Hoa Thi Tran, Eunjoo Nam, Tan Tai Nguyen, Won Jung Yoon, Sungbo Cho, Jungsuk Kim, Keun-A. Chang, Heongkyu Ju

**Affiliations:** Department of Nano-Physics, Gachon University 1342 Seongnam-daero, Sujeong-gu Seongnam-si Gyeonggi-do 461-701 Republic of Korea batu@gachon.ac.kr; GachonBionano Research Institute, Gachon University 1342 Seongnam-daero, Sujeong-gu Seongnam-city Gyeonggi-do 461-701 Republic of Korea; Department of Pharmacology, College of Medicine, Neuroscience Research Institute, Gachon University Incheon 406-799 Republic of Korea keuna705@gachon.ac.kr; Department of Materials Science, School of Basic Science, TraVinh University TraVinh City 940000 Vietnam; Department of Chemical and Bioengineering, Gachon University 1342 Seongnam-daero, Sujeong-gu Seongnam-si Gyeonggi-do 461-701 Republic of Korea; Gachon Advanced Institute for Health Science and Technology, Gachon University Incheon 21999 Republic of Korea; Department of Biomedical Engineering, Gachon University Incheon 21936 Republic of Korea; Neuroscience Institute, Gil Hospital Incheon 405-760 Republic of Korea

## Abstract

We present the immunoassay of tau proteins (total tau and phosphorylated tau) in human sera using surface plasmon resonance (SPR) fiber sensors. This assay aimed at harvesting the advantages of using both SPR fiber sensors and a blood-based assay to demonstrate label-free point-of-care-testing (POCT) patient-friendly assay in a compact format for the early diagnosis of Alzheimer's disease (AD). For conducting the assay, we used human sera of 40 subjects divided into halves, which were grouped into AD patients and control groups according to a number of neuropsychological tests. We found that on an average, the concentrations of both total tau and phosphorylated tau proteins (all known to be higher in cerebrospinal fluid (CSF) and the brain) turned out to be higher in human sera of AD patients than in controls. The limits of detection of total tau and phosphorylated tau proteins were 2.4 pg mL^−1^ and 1.6 pg mL^−1^, respectively. In particular, it was found that the AD group exhibited average concentration of total tau proteins 6-fold higher than the control group, while concentration of phosphorylated tau proteins was 3-fold higher than that of the control. We can attribute this inhomogeneity between both types of tau proteins (in terms of increase of control-to-AD in average concentration) to un-phosphorylated tau proteins being more likely to be produced in blood than phosphorylated tau proteins, which possibly is one of the potential key elements playing an important role in AD progress.

## Abstract 2

Blood-based early diagnosis of Alzheimer's disease using a plasmonic fiber sensor that detects immunoreaction of tau proteins.

## Graphical abstract

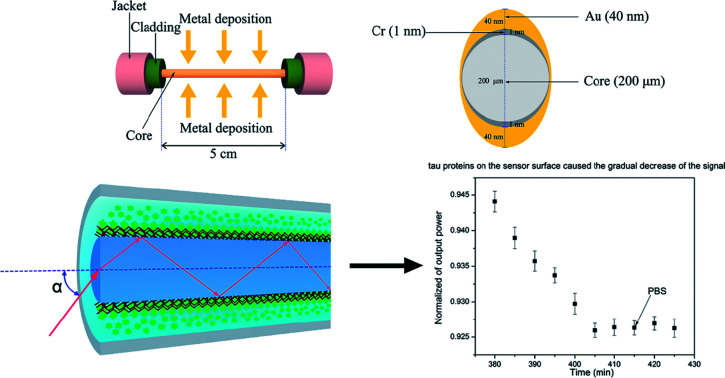

## 1. Introduction

Alzheimer's disease (AD) is the most common type of dementia pathology that occurs in elderly people. As the global population increases in age, the number of people affected will increase. It is estimated that AD is going to affect 115 million individuals worldwide by 2050.^1^ Currently, AD is one of the forefront research subjects in the field of clinical dementia. Two main lesions that form in the brain and thus are responsible for AD include the senile plaques containing the amyloid-beta (Aβ) protein and the neurofibrillary tangles composed of tau proteins.^2–5^ Tau proteins primarily bind to microtubules and help them stabilize. It is known that detachment of tau proteins from the microtubules with neurodegeneration of the senile plaques and neurofibrillary tangles could be invoked to explain the AD-caused dementia.^4,6,7^ Since neurodegenerative disorder is unremitting and progressive, effective methods for the early diagnosis of AD are necessary before the lesions become too severe to cure. Time-, labor- and cost-effective early identification of AD shall thus positively affect the relevant drug therapy and contribute to reducing its associated burden.

Screening of biomarkers for AD has been conducted for the past decades. Numerous potential biomarkers are under investigation, among which candidate proteins, namely, tau and Aβ proteins have been considered as key biomarkers for AD screening.^8–11^ In particular, numerous studies have determined the concentration level of tau proteins in brain or cerebrospinal fluid (CSF) and have demonstrated that tau levels are higher in AD cases than in healthy controls.^12–16^ The difficulty, high cost, and invasiveness associated with obtaining CSF or brain tissue samples may, however, prevent the tau assay from being run in a timely fashion for the early diagnosis of AD. It has also been reported that similar differences possibly existed between concentration levels of tau proteins in the blood of AD patients and those of healthy controls.^17,18^ Such a difference can lead us to expect that development of a blood-based assay will help lower the barrier to opportune AD diagnosis due to the relatively straightforward and cost-effective arrangement of the relevant samples containing tau proteins, as compared to the CSF-based assay. Accordingly, focus has shifted to the blood-based methodology, featured by its relative patient friendliness in collecting diagnostic samples.^17–29^

To detect tau concentration levels in blood, the techniques of single-molecule array (SIMOA),^28,29^ immune magnetic reduction (IMR), and enzyme-linked immunosorbent assay (ELISA) have been utilized. Recent studies using the IMR^17,20,21^ and SIMOA^22,23^ methodologies reported higher levels of tau in AD patient's blood. Moreover, few studies conducted using ELISA have recently demonstrated that there was no distinctly elevated level of tau proteins or that levels even deescalated in AD patient blood as compared to normal controls.^19,24–29^ A commercially available biosensor, which utilized surface plasmon resonance (SPR) in a conventional prism-aided light coupling system, has been used to report that the concentration levels of both the phosphorylated tau and the total tau (which included both un-phosphorylated and phosphorylated ones) contents were higher in AD patient blood than in controls.^18^ It was observed that the different results reported from a number of the aforementioned blood-based assays of tau concentration levels might possibly have been due to the different antibodies used in such assays, which were featured by their characteristic strengths of affinity bonds with the tau proteins in those immunoreactions.

In this study, we present fiber optical methodologies to estimate the concentration levels of both total tau proteins and phosphorylated tau proteins in human sera, which were grouped into AD patients and control groups *via* a number of neuropsychological tests. Prior to the SPR fiber sensing experiment, the grouped sera were tested with ELISA kits, which showed higher concentrations of both phosphorylated tau and total tau proteins on an average, similar to the results obtained by Shekhar *et al.* (2016).^18^

The optical fiber sensor utilized immunoreaction-based SPR as a label-free optical refractometer that needed no fluorophores. SPR is an optical phenomenon, in which characteristic modes of oscillation of conduction electron density are coherently excited by an electromagnetic field of transverse magnetic polarization at the interface between a metal and a dielectric under certain conditions. These resonance conditions can be met *via* evanescent excitation by adjusting the interface-parallel components of the wave vectors of electromagnetic fields to those of the plasmonic fields. The fiber with its cladding replaced by a nanometer thick metal film can provide this SPR condition, through which the fiber core yields sufficiently high wave vectors that meet the relevant phase matching condition.

We coated 40 nm-thick gold (Au) on the surface of the core of a multimode optical fiber for 5 cm along its length. Then, we immobilized antibodies on the Au surface, which enabled immunoreactions specific to the types of tau proteins of interest, *i.e.*, the antibody TAU5 for total tau and the antibody AT8 for phosphorylated tau. We calibrated the changes in a SPR fiber sensor signal with respect to concentrations of pure tau proteins. It was revealed that the limits of detection (LOD) of total tau and phosphorylated tau proteins were 2.4 pg mL^−1^ and 1.6 pg mL^−1^, respectively.

We applied the calibrated sensor to detect concentrations of the total tau and the phosphorylated tau proteins contained in human sera arranged from blood of 40 human subjects aged over 65. The SPR fiber sensor measurements showed that the average concentration of total tau in AD patient sera was 6-fold higher than that in controls, while the average concentration of phosphorylated tau in AD patients was 3-fold higher than that in controls. This indicated that the control-to-AD change in the average concentration of total tau exceeded the corresponding change in that of phosphorylated tau. This inhomogeneity between concentrations of total tau and phosphorylated tau proteins (in terms of the control-to-AD change) revealed higher increase of control-to-AD group sera in un-phosphorylated tau concentrations. This then implied a possibility of different mechanisms that we can attribute to the increase in concentration of tau proteins, accounting for quantitative inhomogeneity between phosphorylated and un-phosphorylated tau proteins in the blood of AD patients.

The fact that the present assay scheme used optical intensity measurements without needing a spectrograph or an angle interrogation setup for SPR-based diagnosis would allow for applications in places where an entire assay system needs to be miniaturized without compromising its SPR-inherent sensitivity. The present methodologies that used the immunoreaction-based SPR fiber sensor with intensity interrogation, therefore, could harvest merits from the fiber-intrinsic easy coupling of light for SPR excitation, the remote diagnosis capability of fibers, and the simplicity of its structure as a blood-based assay. This could thus pave the way to point-of-care-testing (POCT) applications for the early diagnosis of AD and monitoring of its progress in a patient-friendly manner.

## 2. Materials and methods

### 2.1 Human sera

A total of 40 subjects used in this study were supplied by the Gachon University Gil Medical Center, Incheon, Republic of Korea. To categorize cognitive impairment, blood sera from normal control subjects and AD patients were defined by neuropsychological tests that included Mini-Mental State (MMSE), Clinical Dementia Rating (CDR), Clinical Dementia Rating Sum of Box (CDR-SOB), and Global Deterioration Scale (GDS) (Table 1). All subjects were aged over 65 years (40 subjects divided in halves between control and AD groups). A very limited number of subjects who also underwent positron emission tomography (PET) test or SIMOA assay were available for our study though those are established methodologies for AD diagnosis.

**Table 1:** Demographic data of subjects^a^

	Age	Gender	MMSE	CDR	CDR-SOB	GDS
		Male/female				
Controls (*n* = 20)	71.55 ± 1.21	16/4	27.80 ± 0.20	0.48 ± 0.03	0.73 ± 0.08	1.70 ± 0.12
AD (*n* = 20)	74.65 ± 1.27	4/16	20.60 ± 0.84	0.63 ± 0.05	3.15 ± 0.26	3.25 ± 0.12

a Data are presented as mean ± SE.

### 2.2 Ethics

The study was approved by the Ethics Committee and the Institutional Review Board (IRB) of both Gachon University Gil Medical Center (GAIRB2013-264) and Gachon University (1044396-201708-HR-129-01). All study subjects provided informed consent prior to participating in this investigation.

### 2.3 Chemical agents

The specific antibodies TAU5 and AT8 were provided by ThermoFisher (Waltham, MA, USA). Proteins of full length human tau441 and tau [pSp199/202] protein were purchased from Abcam (Cambridge, UK) and USBiological (Salem, MA, USA), respectively. The reagents 11-mercaptoundecanoic acid (11-MUA), *N*-(3-dimethylaminopropyl)-*N*′-ethylcarbodiimide (EDC), *N*-hydroxysuccinimide (NHS), casein-blocking buffer and phosphate-buffered saline (PBS) were acquired from Sigma-Aldrich Co. (St Louis, MO, USA). All agents were diluted in PBS except for 11-MUA, which was diluted in ethanol.

Pellets of Au and chromium (Cr) used for thermal evaporation coating were purchased from iTASCO (Seoul, Korea). To prepare liquid flow cells, polydimethylsiloxane (PDMS), known as Sylgard 184 silicone elastomer kit, was obtained from Dow Corning Corporation (Corning, NY, USA).

Two ELISA kits for screening total tau (MBS022635) and phosphorylated tau (MBS013458) were obtained from MyBioSource, Inc. (San Diego, CA, USA). The capture and detection antibodies used in the MBS022635 kit were mouse monoclonal and rabbit polyclonal to total tau proteins, respectively. The capture and detection antibodies used in the MBS013458 kit were mouse monoclonal and rabbit polyclonal to tau proteins (phosphor S262), respectively.

### 2.4 A SPR fiber sensor head

**Fig. 1:**
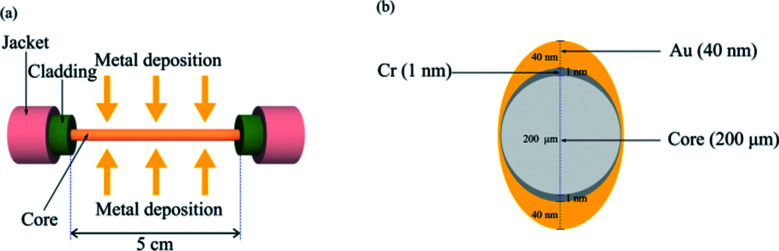
(a) Cr/Au coating on a fiber core; (b) asymmetric cross-section of metal layers coated on the fiber core.

The SPR fiber sensor comprised a multimode fiber (JTFLH-Polymicro Technologies, Molex, Lisle, IL, USA) with its cladding replaced by nanometer-thick Au film for 5 cm along its length as shown in Fig. 1a. This sensor head resulted from a sequential procedure that included the removal of plastic cladding of the fiber along 5 cm and subsequent metal coating by a thermal evaporator on the exposed core. The consecutive evaporation of metals Cr and Au covered the fiber core with 1 nm thick Cr (adhesion) and 40 nm thick Au on one side. This was repeated for coating on the other side of the fiber, expecting an asymmetric coating profile as shown in Fig. 1b. The fiber sensor head was then mounted within a ring-shaped flow cell made of PDMS.

### 2.5 Experimental setup

**Fig. 2:**
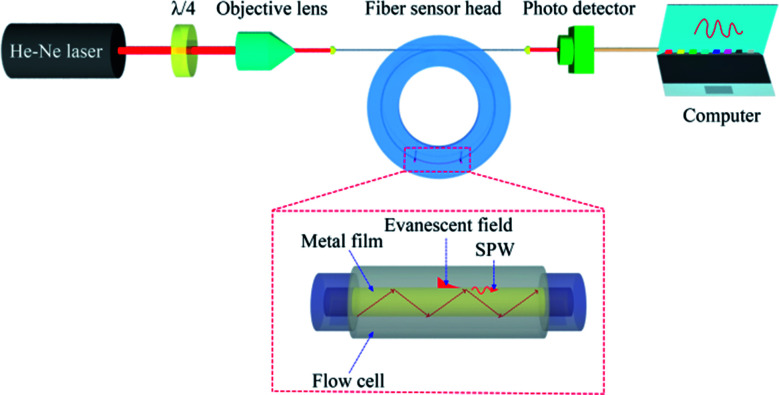
Schematic diagram of optical setup with the SPR fiber sensor for tau protein detection. *λ*/4 denotes a quarter-wave plate.

We used a label-free fiber optical SPR sensor developed recently.^30–33^ A He–Ne laser was used as the light source at 632.8 nm. The laser light that passed through a quarter wave plate (*λ*/4) into a circular polarizer was then coupled into the fiber sensor head by an objective lens of numerical aperture 0.25 as depicted in Fig. 2. The ring-shaped flow cell permitted liquid to flow above the metal surface *via* the inlet and outlet ports. Both the refractive index change in the buffer above the surface and the surface immobilization of biomolecules would cause changes in optical power at the fiber output due to SPR condition changes. The fiber output power was monitored in real time, enabling the kinetic behaviors of the bio-molecular affinity interaction, such as completion of antibody immobilization on the sensing surface and time-dependent antibody–antigen interaction, to be probed and identified.

### 2.6 Immunoassay of tau proteins

**Fig. 3:**
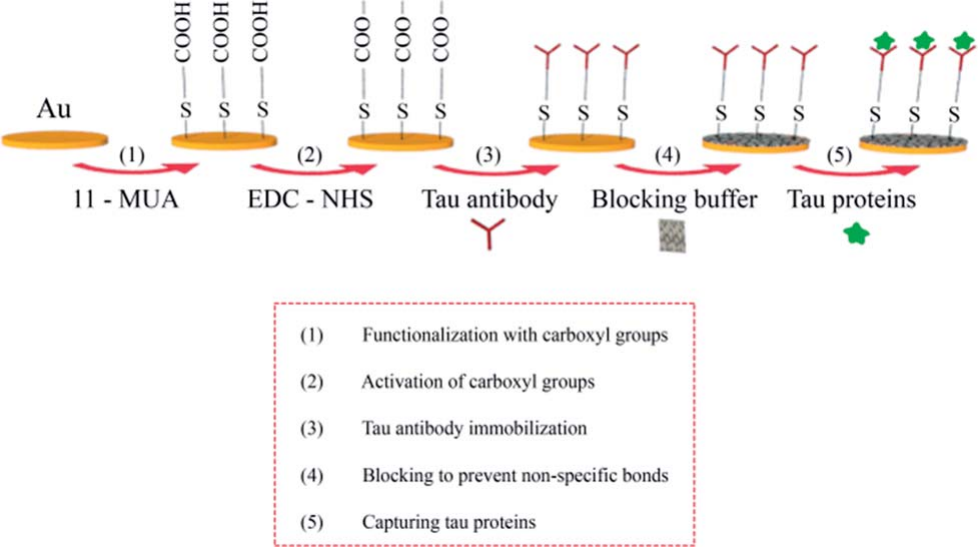
Schematic illustration for the immunoassay of tau proteins on the surface of the SPR fiber sensor.

Fig. 3 shows sensor surface modification for the specific detection of tau proteins. An Au-coated fiber core was functionalized with carboxyl groups using 0.5 mM 11-MUA. The carboxyl groups were subsequently activated with EDC–NHS (0.1–0.4 M). Tau antibodies were then immobilized on the surface, which preceded the injection of casein buffer solution (0.5%), which would cover the remaining spaces on the Au surface to block the nonspecific bonding of subsequently injected molecules. PBS rinsing was used to remove non-specifically bound molecules on the surface. Tau proteins at various concentrations were arranged by different dilution factors (×1000, ×500, ×100), which were injected onto the surface and captured by their corresponding antibodies.

## 3. Results and discussion

### 3.1 Time-dependent signal of the SPR fiber sensor

**Fig. 4:**
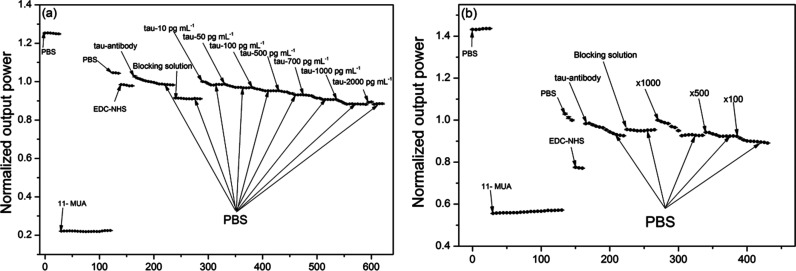
Examples of real-time measurement of the SPR fiber sensor signal (normalized output power) upon injection of a series of biochemical substance. (a) Immuno-detection of pure phosphorylated tau proteins for signal calibration, using its concentrations of 10, 50, 100, 500, 700, 1000 and 2000 pg mL^−1^; (b) immuno-detection of phosphorylated tau proteins present in a human serum of the AD group, after its dilution by 100, 500, and 1000 times.

Fig. 4a shows an example of the real-time sensor response (normalized output power) upon injection of a series of liquids including pure tau proteins at various concentrations (pure phosphorylated tau proteins are used to establish calibration of the sensor signal *versus* tau proteins in this occasion). Each solid circle represents the sensor signal averaged over each time interval of 60 s (the photodetector has data sampling frequency of 20 Hz). The sensor signal change was normalized by the baseline signal, which was obtained by rinsing the surface with PBS buffer immediately prior to injection of each tau protein of given concentration.

The subsequent injection of a series of liquids such as 11-MUA, PBS, EDC–NHS, the antibody (AT8), the blocking agent, and tau proteins induced signal changes *via* changes in both the bulk refractive index and the surface index. It was found that an increase in these effective indices above the sensing surface reduced the sensor output power as a consequence of the SPR condition change. This indicated that as more molecules immobilized on the sensing surface or liquid buffer medium of higher index filled the space near the surface, the metal–dielectric surface structure came closer to the plasmonic resonance and maximized attenuation of the optical power of light propagating in the fiber. For instance, injection of 11-MUA decreased the signal due to its index (1.366) being greater than that of PBS (1.335).

It was also observed that antibody injection increased the signal abruptly due to its buffer solution index being smaller than that of EDC–NHS. However, the surface immobilization of antibodies gradually decreased the signal over time due to the effective index being enhanced by gradual immobilization. The pattern of this type of gradual decrease in signal was observed from points of injection of all concentrations of tau proteins as shown in Fig. 4a. This indicated that the present fiber sensor could be sensitive to effective index change in the region above the Au surface, characterized by decay depth of the SPR evanescent field. These effective index effects can be derived from contributions of the bulk index and from those of the index of surface-immobilized layers.

It should be noted that the consecutive injections of tau protein concentrations required us to re-estimate both its resultant concentration at the injection point and its resultant signal change. For instance, let us assume that we observe signal change Δ*P*_1_ upon injection of 10 pg mL^−1^ tau protein and further change ΔP_2_ upon subsequent injection of 50 pg mL^−1^ tau protein. Then, it is estimated that the concentrations of 10 pg mL^−1^ and 60 pg mL^−1^ (=10 pg mL^−1^ + 50 pg mL^−1^) induce signal changes of Δ*P*_1_ and Δ*P*_1_ + Δ*P*_2_, respectively, taking into account the resultant concentration at the injection point.

Similar to phosphorylated tau protein assay shown in Fig. 4a, we repeated real-time measurements of the sensor signal using pure total tau proteins and the corresponding antibody (TAU5) with another sensor head to obtain the relevant calibration of the signal change *versus* concentration. A method for calibrating the signal change induced by immunoreaction of pure total tau and pure phosphorylated tau proteins *via* nonlinear fitting will be described in the next section. This method takes into account the elliptical nature of the cross-sectional profile of SPR metal coated on the fiber core.

Moreover, determination of the concentrations of total tau and phosphorylated tau in human sera required us to repeat the sensor signal measurement in real time using a series of liquids that included the corresponding antibodies (either TAU5 or AT8) and human sera diluted by factors of ×1000, ×500, and ×100. Fig. 4b shows one such real-time measurement including immunodetection of the phosphorylated tau proteins present in a human serum (grouped in AD) with the SPR fiber sensor. Each type of tau protein present in one human serum consumed a single SPR fiber sensor head, which was not reusable. Thus, eighty SPR fiber sensor heads were used to obtain the respective eighty graphs of real-time measurements (each similar to those shown in Fig. 4b) considering 40 human subjects and two types of tau proteins probed. The results obtained with the human sera are summarized and discussed in the section on tau concentrations in blood.

### 3.2 Calibration curves

We calibrated the sensor signal change with respect to the concentrations of total tau and phosphorylated tau proteins. This calibration was required to estimate the concentrations of tau proteins present in human sera. For this calibration, we used pure total tau (tau441) and pure phosphorylated tau (pSp199/202) proteins to observe the sensor signal change caused by only immunoreaction of the tau proteins with the corresponding antibodies. The concentration used for calibration ranged from 10 pg mL^−1^ to 2360 pg mL^−1^ of total tau and 10 pg mL^−1^ to 4360 pg mL^−1^ of phosphorylated tau proteins.

**Fig. 5:**
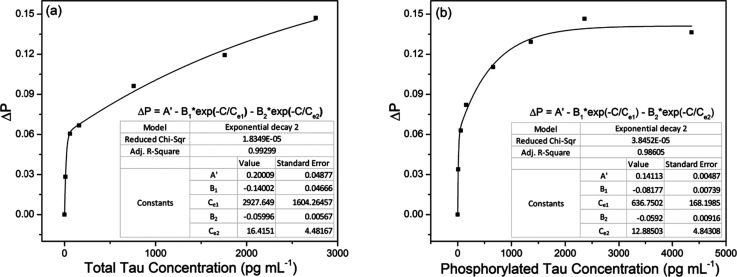
Nonlinear fitting for calibration of the normalized signal change (Δ*P*) *versus* tau concentration (*C*). (a) Normalised Δ*P versus* concentration of total tau (with TAU5 antibody) ranging from 0 to 2360 pg mL^−1^; (b) normalized Δ*P versus* concentration of phosphorylated tau (with AT8 antibody) ranging from 0 to 4360 pg mL^−1^.

Fig. 5a and b show the normalized sensor signal change (Δ*P*) *versus* total tau concentration and that *versus* phosphorylated tau concentration, respectively. The signal change was normalized with respect to the signal at the starting point, at which the signal change began. We achieved nonlinear fits to measurement (represented by solid lines), considering the elliptical profile of the cross-section of the SPR metal layer coated on the fiber core. It was estimated that the SPR fiber sensor had total tau LOD of 2.4 pg mL^−1^ (0.53 fM) and phosphorylated tau LOD of 1.6 pg mL^−1^ (1.3 pM). This indicated that the antibody used to capture total tau proteins (molecular weight of 46 kDa) had stronger affinity than that used for phosphorylated tau proteins (molecular weight of 1.223 kDa).

The SPR evanescent field amplitude that decayed exponentially above the sensing surface would not allow a linear relationship between the sensor signal change and the concentration. Higher concentration of tau proteins that would likely occupy higher regions above the sensing surface would interact with a weaker SPR evanescent field with a consequence of inducing smaller changes in the sensor signal. This gave rise to the nonlinear relationship of Δ*P versus* tau concentration (*C*) shown in Fig. 5a and b.

To fit the measurement, we used the nonlinear function of the form

1 Δ*P* = *A* − *B*_1_ exp(−*C*/*C*_e1_) − *B*_2_ exp(−*C*/*C*_e2_),

where *A*, *B*_1_ and *B*_2_ are positive constants obtainable by fitting. Two exponential functions were introduced to reflect two effective depths, over which the surface plasmon evanescent fields decayed in the two directions normal to the surface of coated metal with elliptical cross-sectional profile (Fig. 1b). Thus, *C*_e1_ and *C*_e2_ that were also used as fitting parameters denoted the tau concentrations, above which the number of tau protein molecules interacting with evanescent fields would decrease exponentially. This led to gradual increase in Δ*P* with an increase in *C*. We found that the use of two exponential functions could fit the measurement (Δ*P versus C*) better than a single exponential function, indicating that the asymmetrical profile of the metal coating in the presented sensor could excite surface plasmons effectively under the two SPR conditions.

It is also interesting to note that the non-uniform profile of coated metal could support more fiber optical modes to excite SPR, favouring enhancement of sensor sensitivity.

### 3.3 Tau concentrations in blood

**Fig. 6:**
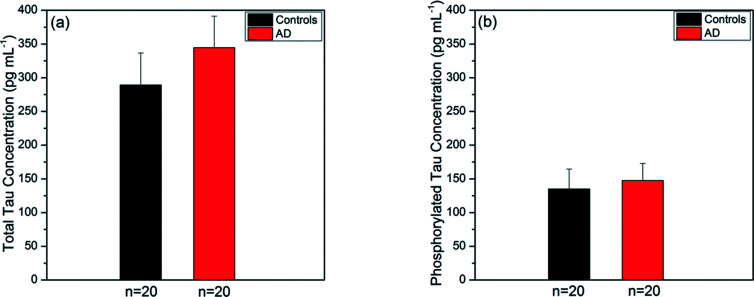
Average tau concentrations of AD and control group sera, measured by ELISA kits (a) total tau protein levels (mean ± SE) were 344.59 ± 46.52 pg mL^−1^ for AD group (*n* = 20) and 289.09 ± 47.53 pg mL^−1^ for control group (*n* = 20); (b) phosphorylated tau protein levels (mean ± SE) were 147.50 ± 25.32 pg mL^−1^ for AD group (*n* = 20) and 134.90 ± 29.48 pg mL^−1^ for control group (*n* = 20).

Prior to experiments with the SPR fiber sensors, we used the ELISA kits (utilizing a sandwiched immunoassay) to measure the tau concentrations present in the same human sera that would be used for the present sensor. We took their averages over AD and control groups for each type of tau protein. The ELISA kits were known to have LOD of 2.0 pg mL^−1^ and 1.0 pg mL^−1^ for total tau and phosphorylated tau, respectively. The average concentration of total tau over the AD group was 344.59 ± 46.52 pg mL^−1^ (mean ± SE) as shown in Fig. 6a. This was higher than the average over the control group (289.09 ± 47.53 pg mL^−1^ (mean ± SE)). Fig. 6b provides the average concentrations of phosphorylated tau proteins in the AD and control groups, which were 147.50 ± 25.32 pg mL^−1^ and 134.90 ± 29.48 pg mL^−1^, respectively. Similarly, the average over the AD group was higher than that over the control group.

In summary, tau protein immunoassay by ELISA method showed that the average concentration of human serum tau proteins in the AD group was higher than that in the control group for both types of tau proteins. The average concentration of total tau increased by a factor of 1.2, while that of phosphorylated tau increased by a factor of 1.1 as the subjects changed from the AD to control group. The control-to-AD increase in average total tau concentration was slightly larger than that in average phosphorylated tau concentration.

**Fig. 7:**
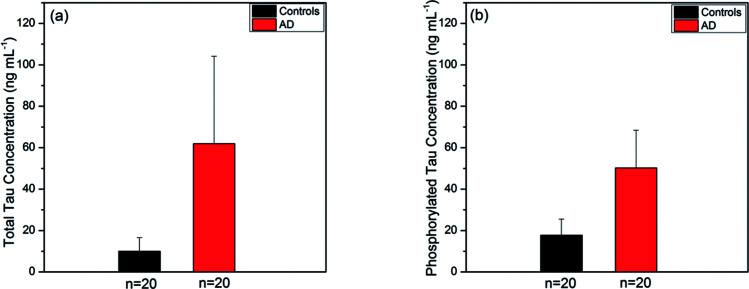
Average of tau concentrations of AD and control group sera, measured by SPR fiber sensors (a) total tau protein levels (mean ± SE) were 61.91 ± 42.19 ng mL^−1^ for AD group (*n* = 20) and 9.99 ± 6.61 ng mL^−1^ for control group (*n* = 20); (b) phosphorylated tau protein levels (mean ± SE) were 50.25 ± 18.17 ng mL^−1^ for AD group (*n* = 20) and 17.74 ± 7.86 ng mL^−1^ for control group (*n* = 20).

We applied the present SPR fiber sensor to human sera of 40 subjects, obtained sensing measurement data and summarised the subsequent analyses of each type of tau protein for comparison between AD patients and controls. Fig. 7a and b show the tau concentration averages of human blood subjects (20 AD subjects and 20 controls). It was revealed that the average concentration of total tau in AD patients (61.91 ± 42.19 ng mL^−1^) was nearly 6-fold higher than that in controls (9.99 ± 6.61 ng mL^−1^) as illustrated in Fig. 7a. It was also found that the average phosphorylated tau concentration was 3-fold higher in AD patient blood (50.25 ± 18.17 ng mL^−1^) than in the control (17.74 ± 7.86 ng mL^−1^) as shown in Fig. 7b. Unlike the ELISA kit results mentioned above, the SPR fiber sensor measurement produced inhomogeneity between total tau and phosphorylated tau proteins in terms of control-to-AD increase in average concentration. This was partly attributed to the use of antibodies for both types of tau proteins in the ELISA kits, which were different from those used in the SPR fiber sensor presented herein, particularly in terms of affinity strength. The inhomogeneity may imply that different mechanisms were involved with the production of phosphorylated and un-phosphorylated tau proteins in blood. It was thus possibly conjectured that un-phosphorylated tau proteins were more likely to be produced in AD sera and this is considered one of the potential key elements that played a vital role in AD progress.

It was noted that detection of both types of tau proteins relied on their respective different antibodies immobilized on the Au surface of the SPR fiber sensor. Total tau LOD (in mass coverage) larger than that of phosphorylated tau indicates that the antibody TAU5 had weaker bio-affinity than AT8. This may induce a larger variance in total tau detection than in phosphorylated tau as shown in Fig. 6a and b. This large variance can be reduced by using higher affinity strength antibodies, thus permitting steadier measurements of total tau proteins.

It was also found that the use of different antibodies, which would have inherently different strengths of affinity to the corresponding tau proteins, would not allow us to estimate the concentration of un-phosphorylated tau proteins simply by subtracting the phosphorylated tau concentration from that of total tau concentration. Nonetheless, it is still valid to evaluate how the tau concentration changed between AD and control subjects for a given type of tau protein as far as the same type of antibody (and same concentration of antibody) was used.

It should be mentioned that the limited number of available blood samples of human subjects that had undergone psychological tests disabled us from obtaining reliable results using a statistical probe such as a *t*-test.

## 4. Conclusion and outlook

We demonstrated a SPR fiber sensor for blood-based immunoassay without fluorophores (a label-free sensor) for detecting tau proteins, which are possible biomarkers for AD dementia. This immunoassay detected total tau proteins and phosphorylated tau proteins with LODs of 2.4 pg mL^−1^ and 1.6 pg mL^−1^, respectively. The SPR fiber sensor head presented herein had an Au film about 40 nm thick coated on the core of a multimode optical fiber along 5 cm in length. Unlike conventional prism-aided SPR excitation, this sensor device allowed easy excitation of SPR in a compact format without compromising sensitivity, enabling the label-free sensitive immunoassay to detect tau proteins present in human blood in the POCT mode.

We applied the present sensors to detect total tau and phosphorylated tau proteins contained in human blood of 40 subjects, divided into halves, each for AD and control groups. It was revealed that on an average, AD serum had higher concentration than the control serum for both types of tau proteins. In particular, the control-to-AD group incremental change in average concentration of total tau proteins exceeded that of phosphorylated protein. This presumably indicated that un-phosphorylated tau proteins, possibly considered a potential key element playing an important role in AD progress, were more likely to be produced in AD patient blood.

The present SPR fiber immunoassay for blood-based tau protein detection can find use in on-demand applications in the POCT mode for the early diagnosis of AD dementia by harvesting potential advantages of the blood-based assay device such as remote sensing, device compactness with sufficient sensitivity, miniaturization suited for multiplexed assay, and patient friendliness in collecting diagnostic samples.

## Conflicts of interest

The authors declare no conflict of interest.

## Supplementary Material
